# Evaluation of qPCR for Quantifying *Escherichia coli* and Tetracycline Resistance Genes in Pig Caecal and Pork Skin Samples: A Comparison with Culture-Based Method

**DOI:** 10.3390/antibiotics15090818

**Published:** 2026-08-23

**Authors:** Tina Birk, Annette Nygaard Jensen, Tina Beck Hansen

**Affiliations:** 1 Department of Technology, University College Copenhagen, Sigurdsgade 26, 2200 Copenhagen, Denmark; 2 Research Group for Food Microbiology and Hygiene, National Food Institute, Technical University of Denmark, Henrik Dams Allé, 2800 Kgs. Lyngby, Denmark

**Keywords:** antibiotics, *E. coli*, *Enterobacteriaceae*, real-time qPCR, *tetA*/*tetB*, *E. coli uidA*

## Abstract

**Background/Objectives:** Rapid surveillance tools are needed to monitor antimicrobial-resistant bacteria in the food chain. This study evaluated whether qPCR-based quantification of tetracycline resistance genes could replace culture-based quantification of tetracycline-resistant *Escherichia coli* in pig caecal content and carcass skin swabs. **Methods:** Caecal content (*n* = 89) and skin swab samples (*n* = 68) were collected across four Danish abattoirs and analysed by both qPCR and Petrifilm cultivation. Method comparisons were restricted to samples within the quantifiable range of Petrifilm. *E. coli* concentrations (*uidA* gene) and *tetA*/*tetB* gene concentrations were compared with Petrifilm counts of *E. coli* and culturable tetracycline-resistant *E. coli*. Method agreement was evaluated using paired *t*-tests, Bland–Altman analysis, and Spearman correlation. **Results:** In caecal samples (*n* = 84), no significant difference in mean log *E. coli* concentration was observed between *uidA* qPCR and Petrifilm counts (*p* = 0.33). However, Bland–Altman analysis showed poor agreement at the individual-sample level, with limits of agreement ranging from −1.09 to 1.21 log_10_ CFU/g. In skin swab samples (*n* = 43), qPCR systematically overestimated *E. coli* counts compared with cultivation, with a significant bias of 0.75 log_10_ CFU/1400 cm^2^ (*p* < 0.001). No significant correlation was observed between *tetA*/*tetB* and culturable tetracycline-resistant *E. coli* in caecal (*r_s_* = 0.091, *p* = 0.44) or skin swab samples (*r_s_* = 0.13, *p* = 0.48). **Conclusions:** qPCR may be useful for population-level surveillance of tetracycline resistance genes in pig caecal samples but cannot replace culture-based methods for accurate quantification of tetracycline-resistant *E. coli* in individual samples.

## 1. Introduction

Antimicrobial-resistant bacteria represent a major threat to the treatment of human infections [1]. The use of antimicrobial agents in food-producing animals may contribute to this burden by selecting for resistant bacteria and resistance genes that can spread from animal reservoirs to humans through the food chain [2,3,4]. Recent One Health surveillance studies have further highlighted the transfer of antimicrobial resistance genes between humans, animals and the environment, emphasizing the need for integrated surveillance approaches [5].

In Danish pig production, aminoglycosides, macrolides, penicillins, and tetracyclines are among the most commonly used antimicrobial classes [6]. Within the Danish Integrated Antimicrobial Resistance Monitoring and Research Programme (DANMAP), surveillance of antimicrobial resistance in animals includes indicator bacteria such as *Enterococcus* spp. and *Escherichia coli* [7]. In *E. coli* isolated from pig caecal samples, resistance to tetracycline was detected in 35% of isolates based on minimum inhibitory concentration (MIC) testing [7].

In Gram-negative bacteria, tetracycline resistance is commonly mediated by *tet* genes located on mobile genetic elements such as transposons and plasmids, facilitating horizontal transfer between bacteria of the same or different species, including potential pathogens [8,9]. Transmission of resistance genes between animal and human reservoirs has been suggested by the detection of identical resistance genes and plasmids in isolates from both sources [2,10,11]. Recent antimicrobial resistance surveillance approaches increasingly combine molecular methods with conventional culture-based methods to improve surveillance [5]. Quantification of resistance genes in animal samples may therefore complement culture-based surveillance and improve our understanding of antimicrobial resistance dissemination between reservoirs. Experimental studies have further demonstrated transmission of tetracycline resistance determinants between antimicrobial-treated and untreated pigs [12]. Tetracyclines are among the most heavily used antimicrobial classes in Danish pig production, and resistance to tetracycline is correspondingly common. In Danish pigs, *E. coli* isolates *tetA* and *tetB* are the predominant tetracycline resistance genes, accounting for 43% and 36% of tetracycline resistance determinants, respectively [6]. These genes were therefore selected as targets for qPCR-based quantification.

Previous studies have compared qPCR and culture-based methods for *E. coli* or related bacteria in other livestock matrices, including bovine faeces [13] and antibiotic resistance genes in swine faeces [14]. A similar study using Bland–Altman analysis has also compared qPCR and plate counts for *Campylobacter jejuni* in poultry neck-skin samples [15]. However, to our knowledge, no study has directly compared qPCR and culture-based quantification of both *E. coli* and tetracycline resistance genes in pig caecal content and carcass skin swab samples, which represent matrices of direct relevance to food-chain surveillance of antimicrobial resistance.

Because complex sample matrices may affect qPCR performance, an initial method-comparison step was included to assess whether qPCR-based estimates of *E. coli* concentration were comparable with culture-based enumeration. For this purpose, the *E. coli*-specific *uidA* gene, encoding β-glucuronidase, was used as the qPCR target [16]. It remained unclear whether qPCR-based quantification would be suitable primarily for population-level surveillance, for individual-sample quantification, or both. The overall aim of this study was to evaluate a real-time qPCR approach for quantifying *tetA* and *tetB* in pig caecal content and carcass skin swab samples and to assess its potential as a rapid alternative to culture-based quantification of tetracycline-resistant *E. coli*.

## 2. Results and Discussion

By quantifying specific resistance genes associated with acquired resistance, it is possible to exclude contributions from naturally intrinsic resistant bacteria, which are not the primary focus of public health monitoring. Current surveillance programmes for monitoring antimicrobial resistance in bacteria rely on traditional culture-based methods combined with phenotypic susceptibility testing or whole-genome sequencing (WGS) of bacterial isolates [7]. The qPCR method therefore offers a rapid alternative approach for investigating the occurrence and dissemination of tetracycline resistance compared to these culture-based methods, which require several days to complete.

**Figure 1:**
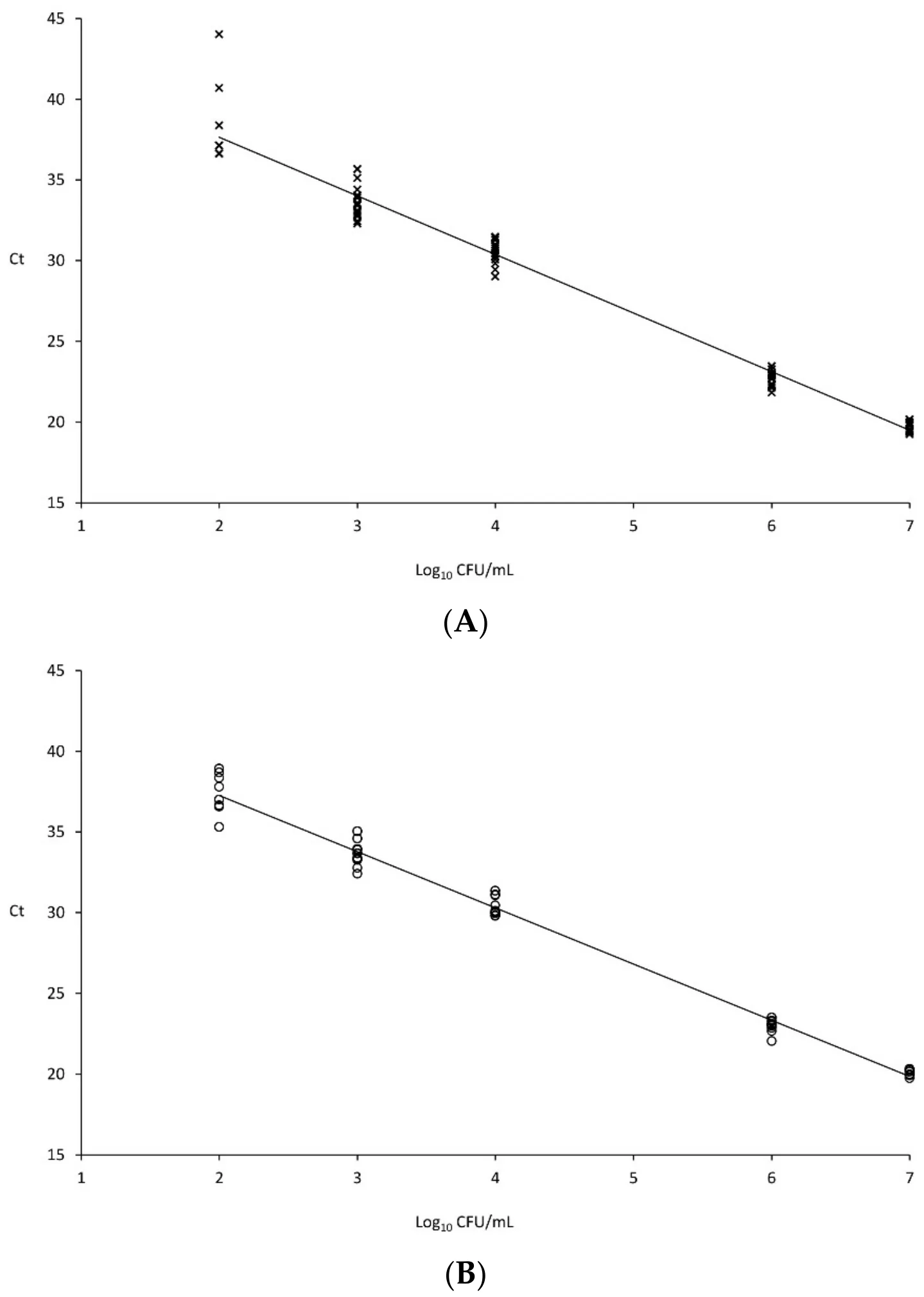

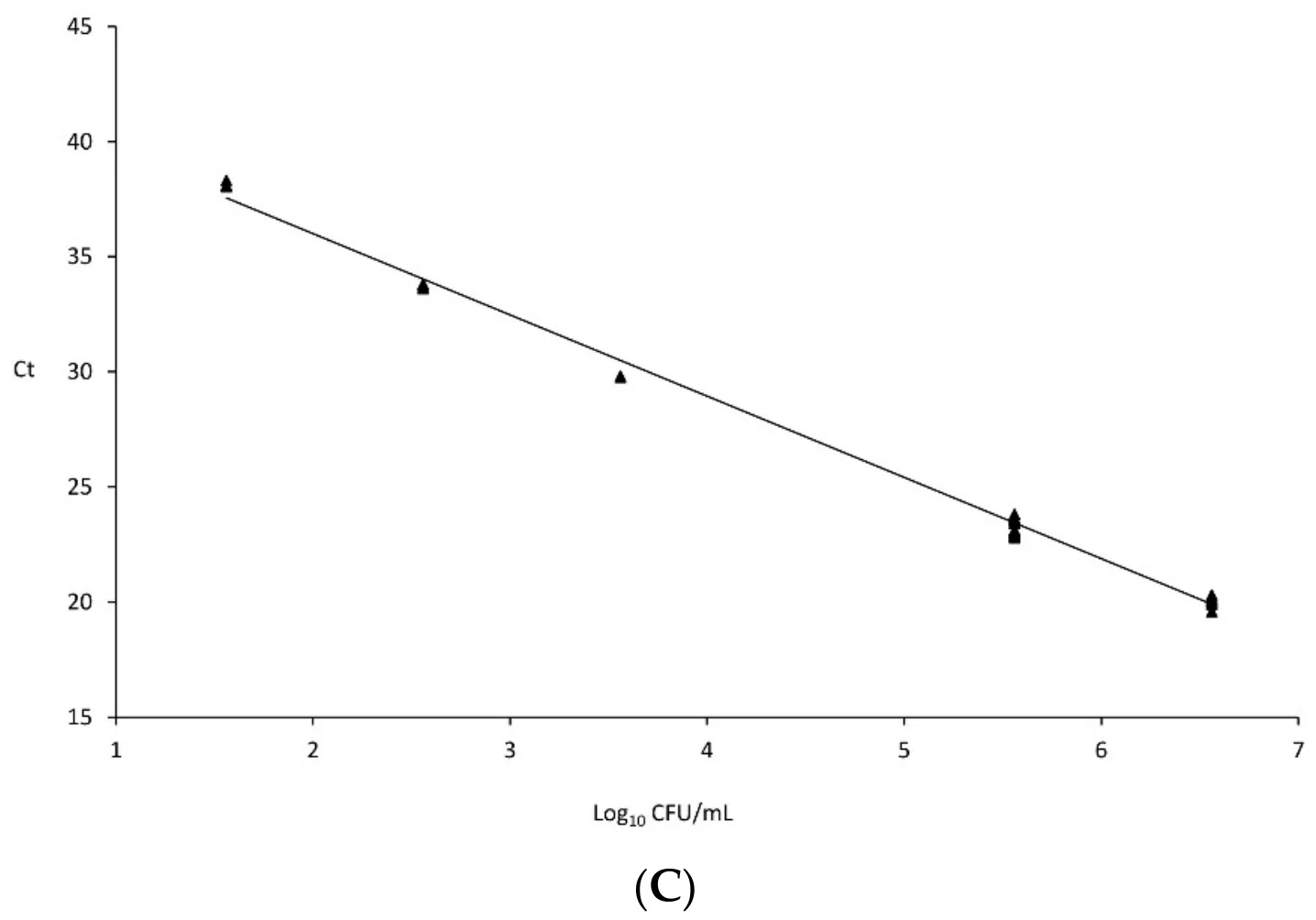
qPCR standard curves and linear regression analyses for the genes (**A**) *uidA*, (**B**) *tetA*, and (**C**) *tetB*. The linear regression equations and coefficients of determination (R^2^) were *uidA*, y = −3.6317x + 44.911 (R^2^ = 0.975); *tetA*, y = −3.4777x + 44.213 (R^2^ = 0.988); and *tetB*, y = −3.5272x + 43.046 (R^2^ = 0.996).

The standard curves are shown in Figure 1; the linear regression equations and their corresponding coefficients of determination (R^2^ = 0.975, 0.988, and 0.996) are shown in the figure legend. The range of Ct for *uidA* spanned from 19.3 to 44.0 (2–7 log_10_ CFU/mL), for *tetA* 19.8–38.9 (2–7 log_10_ CFU/mL), and for *tetB* 19.6–38.3 (1.6–6.6 log_10_ CFU/mL). A limit of detection was not determined for any of the three genes, and the reported ranges reflect the tested standard curve range. Ct values at the lowest standard curve concentration showed considerable variability between replicates for *uidA* (36.4–44.0, 2 log_10_ CFU/mL, *n* = 11) and, to a lesser extent, *tetA* (35.2–38.9, 2 log_10_ CFU/mL, *n* = 14). For *tetB*, only three replicates were available at the lowest concentration (38.0–38.3, 1.6 log_10_ CFU/mL). Quantification of low *uidA* and, to some degree, *tetA* concentrations close to their lowest tested standard points should therefore be interpreted with some caution. Intra-assay variability, assessed as the mean percentage relative standard deviation (%RSD) between Ct values of duplicate reactions in standards, was 1.2% for *uidA*, 1.2% for *tetA*, and 1.1% for *tetB*. Inter-assay variability, assessed using standard curve replicates across different runs, was 3.3% for *uidA*, 1.3% for *tetA*, and 1.0% for *tetB*. During validation of the qPCR assays, the amplification efficiencies of standard curves for the *uidA* (*E. coli*), *tetA*, and *tetB* genes were 89% (95% CI: 85 to 93%), 94% (95% CI: 91 to 97%), and 92% (95% CI: 89 to 95%), respectively. These results demonstrate good amplification efficiency for all three genes.

**Figure 2:**
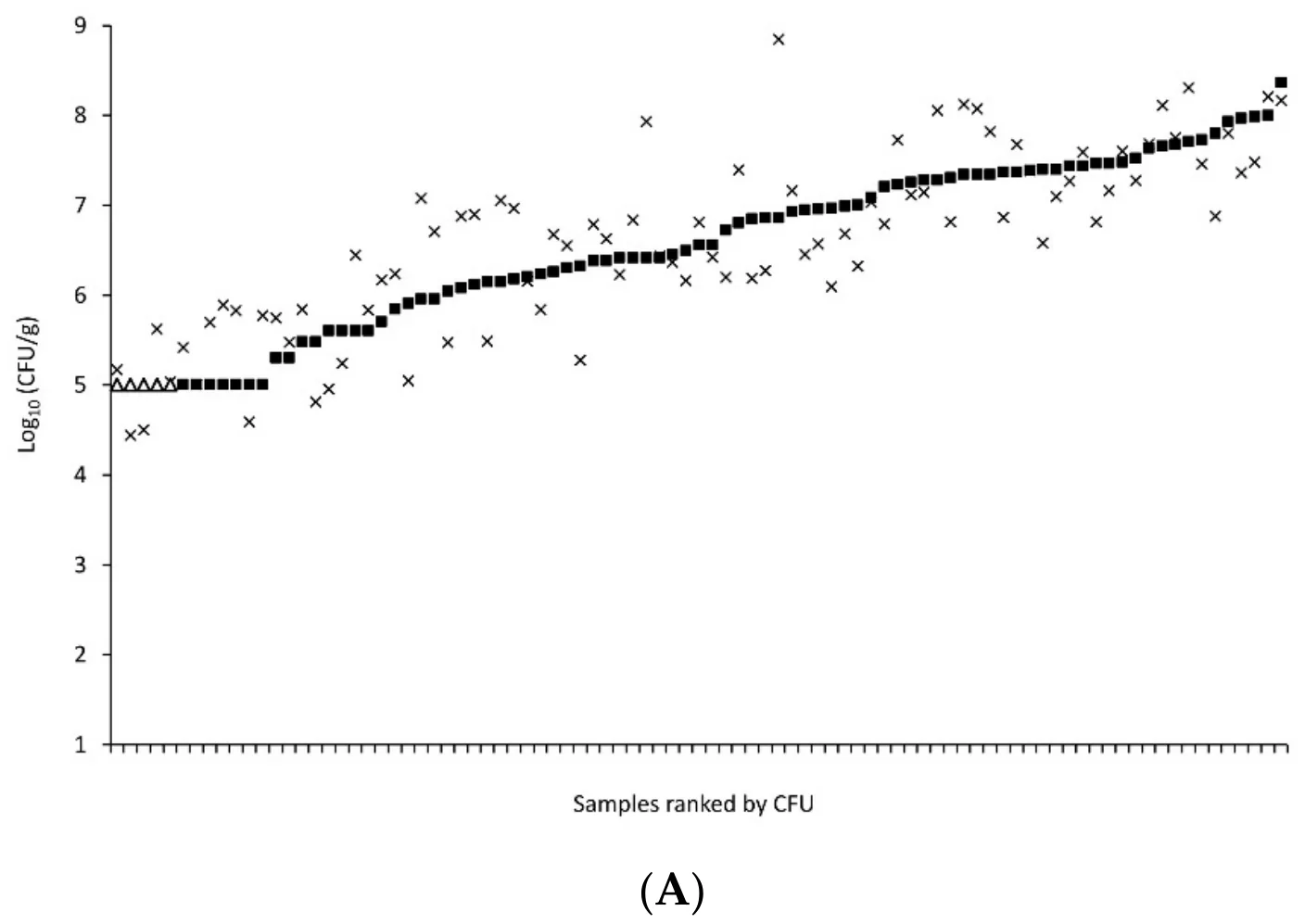

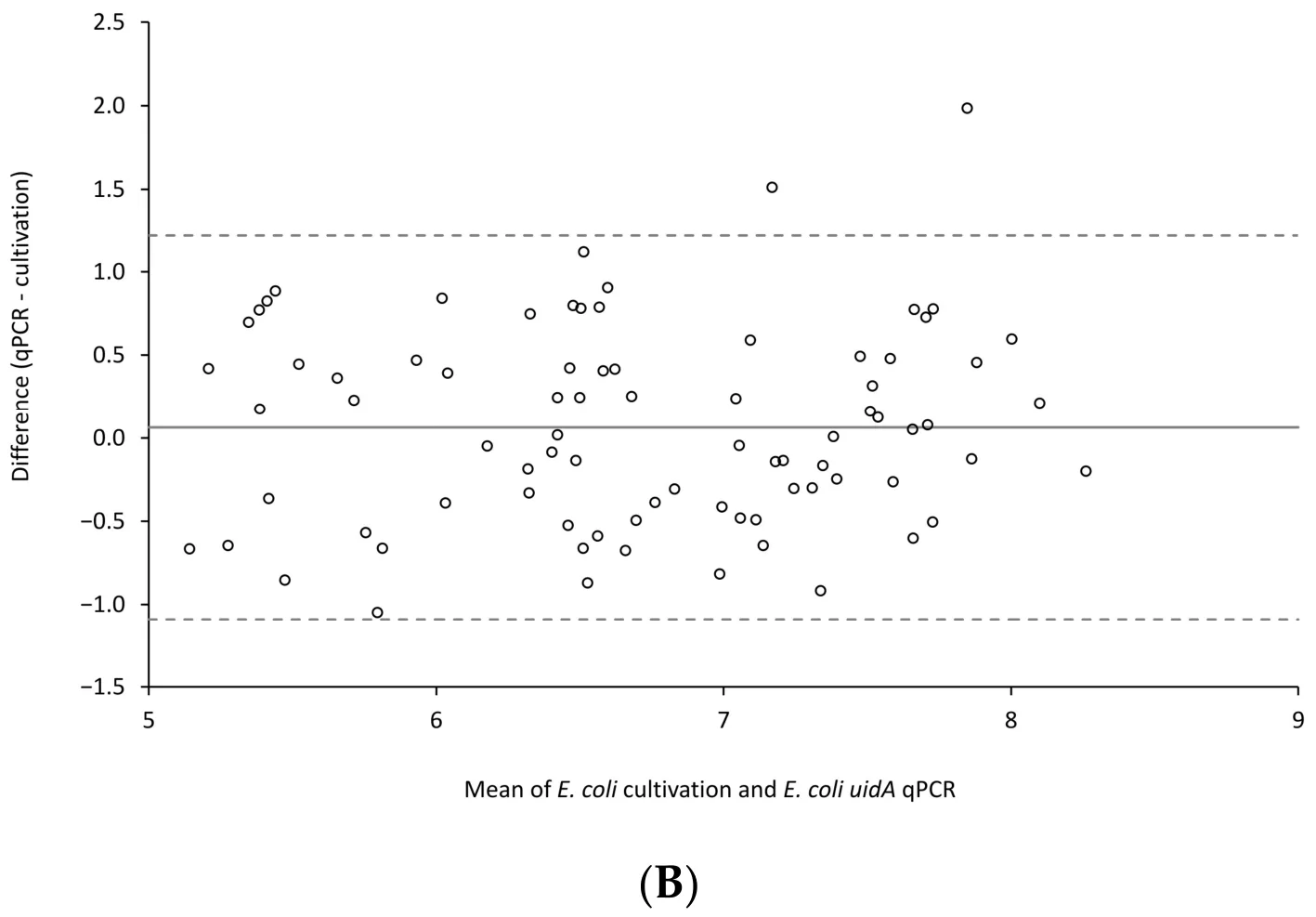
(**A**) Comparison of *E. coli* CFU cultivation concentrations (△■) with *E. coli uidA* qPCR concentrations (×) on individual sample level for caecal samples (*n* = 89). The CFU values are plotted in ascending order with their corresponding qPCR values. Five caecal cultivation results were < 5 log_10_(CFU/g) (△). (**B**) Bland–Altman plot (*n* = 84) comparing the paired log-transformed concentrations log_10_(CFU/g). The *X*-axis represents the mean concentration of the two methods, and the *Y*-axis represents the difference (cultivation minus qPCR). The solid horizontal line indicates the mean bias, and the dashed lines indicate the 95% limits of agreement.

**Figure 3:**
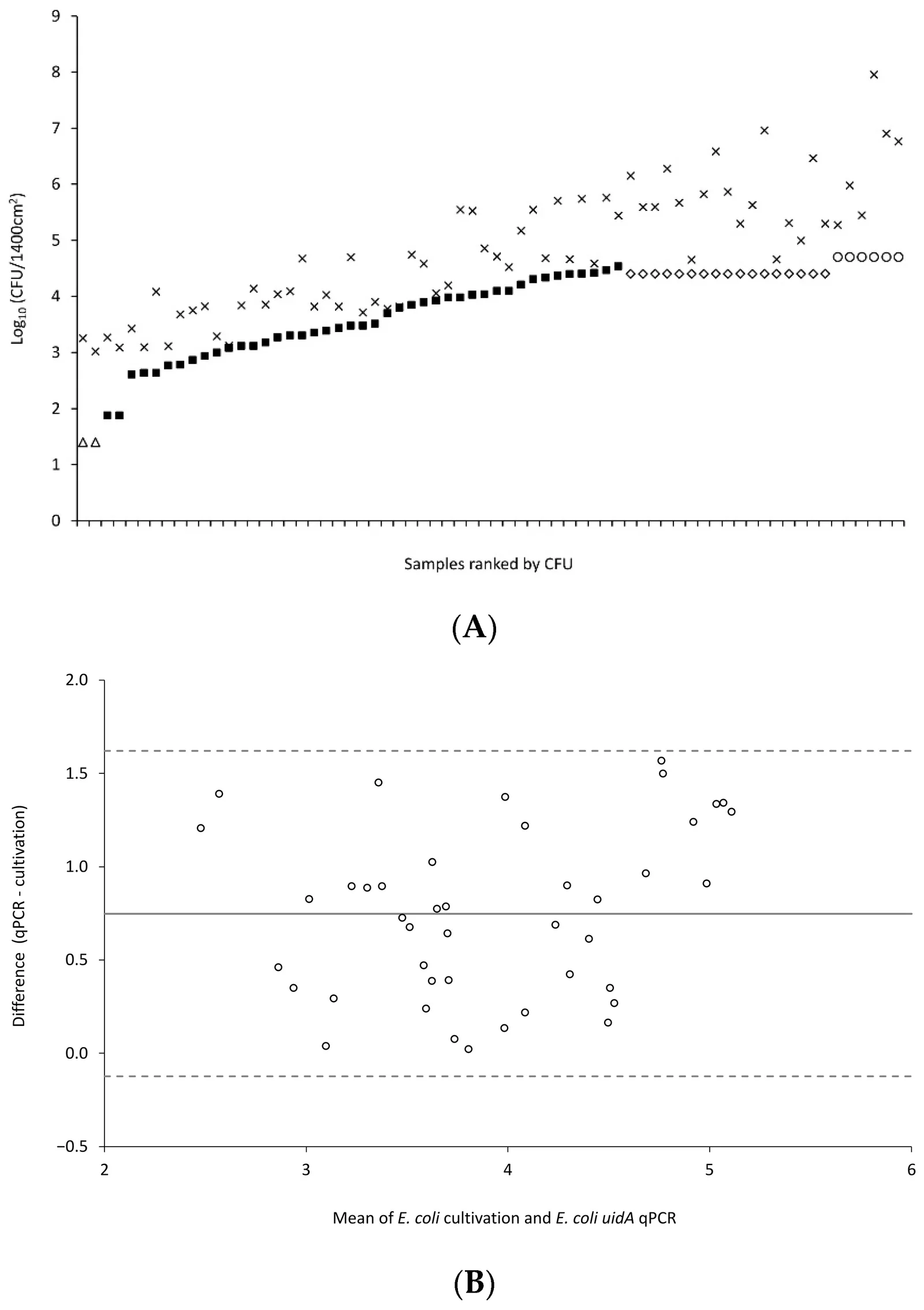
(**A**) Comparison of *E. coli* CFU cultivation concentrations (△■◇◯) with *E. coli uidA* qPCR concentrations (×) on individual sample level for skin swab samples (*n* = 68). The CFU values (log_10_(CFU/1400 cm^2^)) are plotted in ascending order with their corresponding qPCR values where two skin swab samples were <1.4 (△), 17 samples were >4.4 (◇), and six samples were >4.7 (◯). (**B**) Bland–Altman plot (*n* = 43) comparing the paired log-transformed concentrations log_10_(CFU/1400 cm^2^). The *X*-axis represents the mean concentration of the two methods, and the *Y*-axis represents the difference (cultivation minus qPCR). The solid horizontal line indicates the mean bias, and the dashed lines indicate the 95% limits of agreement.

To evaluate the performance of the qPCR assay for naturally contaminated matrices, the concentrations of *E. coli* in caecal and skin swab samples obtained by plating on Petrifilm (CFU/g or CFU/1400 cm^2^) were compared with *E. coli* concentrations determined by qPCR targeting the *uidA* gene. For this comparison, the samples were ranked on the x-axis by ascending Petrifilm plate counts for both the 89 caecal samples (Figure 2A, △ and ■) and the 68 skin swab samples (Figure 3A, △ ■ ◇ ◯). The corresponding qPCR-derived *E. coli* concentration for each individual sample was then plotted alongside these values (Figure 2A and Figure 3A, ×).

A non-significant difference in mean concentrations does not necessarily indicate agreement between methods at the individual-sample level; Bland–Altman analysis was therefore used to assess agreement on a sample-by-sample basis. For caecal samples, mean log *E. coli* concentrations were 6.70 ± 0.92 log_10_ CFU/g by *uidA* qPCR and 6.63 ± 0.88 log_10_ CFU/g by Petrifilm plate counts. A paired *t*-test (*n* = 84) indicated that the mean difference between methods was not significantly different from zero (*p* = 0.33; Figure 2A). Consistent with this, a Bland–Altman analysis comparing the two methods at the individual-sample level (*n* = 84) estimated a mean difference (bias) of 0.063 log_10_ CFU/g (SD = 0.59 log_10_ CFU/g; 95% CI: −0.062 to 0.19 log_10_ CFU/g) (Figure 2B). Since this interval includes zero, it confirms the absence of a systematic over- or underestimation between the methods with respect to the mean *E. coli* concentration. Nevertheless, the 95% Limits of Agreement (LoA) had upper and lower limits of 1.22 log_10_ CFU/g (95% CI: 1.00 to 1.44 log_10_ CFU/g) and −1.09 log_10_ CFU/g (95% CI: −1.31 to −0.87 log_10_ CFU/g), respectively. To evaluate whether the two methods can be considered equivalent, the threshold of 0.5 log_10_ for acceptable method bias from ISO 16140 can be used [17]. Comparing this threshold with the observed bias of 0.063 log_10_ CFU/g shows that the two methods agree well at the population level; however, no formal acceptability criterion exists for LoA in this context, and we therefore apply the same 0.5 log_10_ threshold here as a conservative benchmark for agreement at the individual-sample level. The observed difference between the cultivation and qPCR methods at the individual sample level was <0.5 log_10_ units for 50 out of the 84 paired samples, while the remaining 34 samples differed by more than 0.5 log_10_ units. As high *E. coli* levels were expected in the caecal samples, dilutions corresponding to concentrations below 5 log_10_ CFU/g were not plated on Petrifilm; nevertheless, five samples fell below this level (Figure 2A, △). For these five samples, the qPCR *E. coli* estimates remained within 0.6 log_10_ units of the applied 5 log_10_ CFU/g detection limit (Figure 2A, ×).

The overestimation of *E. coli* concentrations by qPCR relative to culture-based methods may be explained by the detection of total DNA within the sample, including DNA from dead cells, sublethally damaged cells, and bacteria in a viable but non-culturable (VBNC) state [18]. Conversely, 16 qPCR samples yielded concentrations that were more than 0.5 log_10_ units lower than those obtained by cultivation. Underestimated concentrations could be due to the presence of PCR inhibitors such as bile salts, fats, and proteins within the faecal matrix [19], which can impair amplification efficiency in qPCR.

Although the estimated *E. coli* log_10_ CFU/g mean difference (bias) was low between qPCR and Petrifilm for pig caecal samples, the wide Limits of Agreement (upper and lower limits of 1.22 and −1.09 log_10_ CFU/g, respectively; Figure 2B) indicate that the qPCR method cannot directly replace cultivation at the individual-sample level. A similar pattern has been reported in bovine faecal samples, where qPCR agreed closely with cultivation under favourable conditions but increasingly overestimated counts under temperature stress [13]. Caecal and skin swab samples collected in the present study were subjected to process-related stress (e.g., temperature changes, mechanical handling). This stress may have induced a shift toward non-culturable cells and could explain the discrepancies observed here, in addition to potential PCR inhibitors, which are often matrix-specific [20]. However, no internal amplification control was included in this study to detect potential PCR inhibition; the role of PCR inhibitors in the observed variation is therefore proposed based on the established literature. At the population level, no significant difference was observed between the mean *E. coli* concentrations determined by qPCR and cultivation in the caecal samples, supporting the potential use of qPCR-based quantification as a fast-screening tool. Although qPCR-based quantification does not fully correspond to culture-based quantification at the individual-sample level, the detection of *E. coli* and associated tetracycline resistance genes by qPCR nonetheless indicates that these samples may carry a risk of horizontal transfer of resistance genes between bacteria [21]. This poses a potential public health concern, as bacteria within the human gut could acquire resistance plasmids through such transfer.

For the 68 skin swab samples, the *E. coli* concentration fell below the lower detection limit of 1.4 log_10_ CFU/1400 cm^2^ on Petrifilm in two samples (Figure 3A, △). Conversely, 17 samples (Figure 3A, ◇) and 6 samples (Figure 3A, ◯) exceeded the upper detection limits of 4.4 and 4.7 log_10_ CFU/1400 cm^2^, respectively. These samples were excluded because no valid culture-based concentration was available for comparison. Therefore, only the 43 samples with cultivation values ranging from 1.4 to 4.4 log_10_ CFU/1400 cm^2^, along with their corresponding qPCR data, were included in the statistical comparison of the two methods (Figure 3A, ■ ×). Statistical analysis using a paired *t*-test (*n* = 43) showed that the mean *E. coli* concentration (log_10_ CFU/1400 cm^2^) estimated by qPCR (4.28 ± 0.79) was significantly higher than that obtained using Petrifilm cultivation (3.53 ± 0.68) (*p* < 0.001). This was further supported by Bland–Altman analysis, which revealed a systematic bias of 0.75 log_10_ CFU/1400 cm^2^ (SD = 0.44 log_10_ CFU/1400 cm^2^; 95% CI: 0.62 to 0.88 log_10_ CFU/1400 cm^2^; Figure 3B), confirming that qPCR systematically overestimates *E. coli* concentrations in skin swab samples compared with Petrifilm cultivation. The upper and lower Limits of Agreement (LoA) were 1.62 log_10_ CFU/1400 cm^2^ (95% CI: 1.39 to 1.85 log_10_ CFU/1400 cm^2^) and −0.12 log_10_ CFU/1400 cm^2^ (95% CI: −0.35 to 0.11 log_10_ CFU/1400 cm^2^), respectively. This corresponds to a total LoA span of approximately 1.74 log_10_ units, which substantially exceeds the predefined acceptable threshold of 0.5 log_10_ units [17]. As for caecal samples, the observed bias itself (0.75 log_10_ CFU/1400 cm^2^) also exceeds the 0.5 log_10_ threshold, indicating that qPCR does not agree with cultivation even at the population level for skin swab samples. Furthermore, the 95% CI for the lower LoA crosses zero, indicating uncertainty as to whether the true lower limit is below zero. In contrast, no bias was observed when comparing qPCR and plate count methods for quantification of *Campylobacter jejuni* on chicken neck skin, but the wide scatter and large standard deviation reported in that study are consistent with the individual-level variability observed in this study [15]. Collectively, these findings could indicate that a substantial number of dead, sublethally stressed, or viable but non-culturable (VBNC) *E. coli* cells are present on the carcass skin and are detected by qPCR.

**Figure 4:**
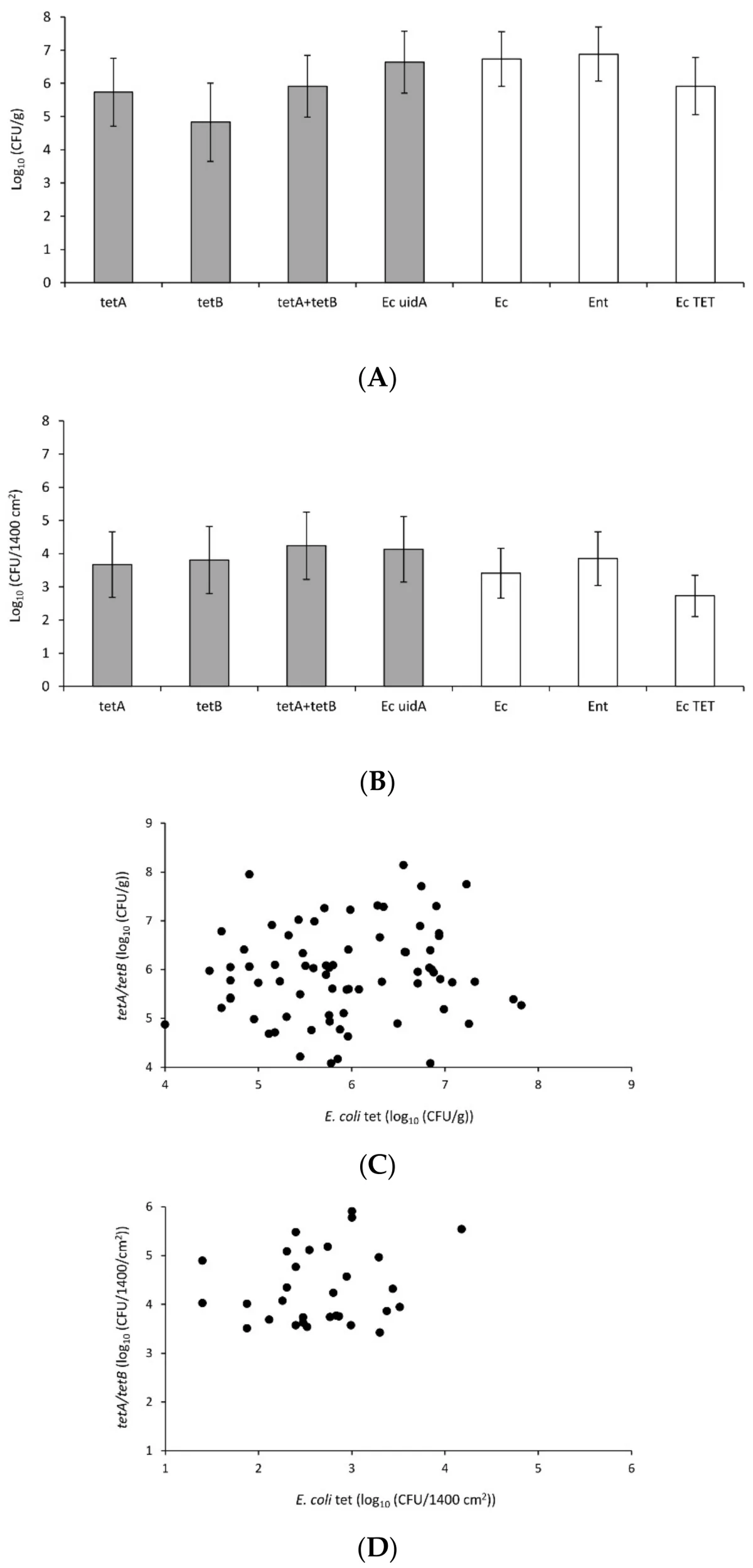
Cultivation data (white columns): *E. coli* (Ec), Enterobacteriaceae (Ent), and tetracycline-resistant *E. coli* (Ec TET); qPCR data (grey columns): *E. coli* uidA gene (Ec uidA) and resistance genes tetA, tetB, and tetA + tetB. Columns represent mean values ± SD. (**A**) Caecal samples (*n* = 75) and (**B**) skin swab samples (*n* = 30). Scatter plots of *tetA/tetB* gene concentrations (qPCR) versus tetracycline-resistant *E. coli* counts (Petrifilm) for (**C**) caecal samples (*r_s_* = 0.091, 95% CI: −0.14 to 0.31, *p* = 0.44) and (**D**) skin swab samples (*r_s_* = 0.13, 95% CI: −0.24 to 0.47, *p* = 0.48); no linear or other systematic pattern was observed in either matrix.

The real-time qPCR method developed for quantifying the tetracycline resistance genes *tetA* and *tetB* was evaluated by comparison with culture-based quantification of tetracycline-resistant *E. coli*. Quantification of *tetA*, *tetB*, and *uidA* genes by qPCR is presented alongside the tetracycline-resistant *E. coli*, *E. coli* and *Enterobacteriaceae* concentrations obtained by plating on Petrifilm (Figure 4). To avoid skewness, only samples containing quantifiable tetracycline-resistant *E. coli* on Petrifilm were included in the statistical analysis, resulting in 75 caecal samples (4.0 to 7.8 log_10_ CFU/g) and 30 skin swab samples (1.4 to 4.2 log_10_ CFU/1400 cm^2^).

For caecal samples (*n* = 75), there were no significant difference in the mean concentration (log_10_ CFU/g) of *tetA*/*tetB* genes (5.91 ± 0.93) determined by qPCR and tetracycline-resistant *E. coli* (5.91 ± 0.86) obtained by Petrifilm (*p* = 0.99) (Figure 4A). The findings indicate that the two methods provide similar estimates of the mean concentration at the population level. This is consistent with findings from a Danish study comparing qPCR and CFU-based quantification of antibiotic resistance genes in swine faeces, which similarly found qPCR more suitable for detecting differences at the population/herd level than individual-level CFU counts [14]. However, a Spearman rank correlation analysis revealed no statistically significant correlation between the two methods on an individual sample level (*r_s_* = 0.091; Figure 4C, see Table 1 for full statistics). This implies that samples with a high concentration of *tetA*/*tetB* genes did not consistently correspond to a high concentration of tetracycline-resistant *E. coli* on Petrifilm, indicating a lack of correlation between qPCR gene quantification and culture-based detection. This discrepancy may partly reflect that *tetA*/*tetB* genes can also be carried by bacterial species other than *E. coli* [22], meaning qPCR detection of these genes does not directly correspond to counts of tetracycline-resistant *E. coli*. Previously, Yu et al. [23] quantified *tet* genes in pig faecal samples using qPCR using SYBR Green, which is less specific than our probe-based qPCR. However, because their study focused exclusively on molecular quantification, no comparison with culture-based enumeration was performed.

For the skin swab samples, (*n* = 30) the mean qPCR concentration (log_10_ CFU/1400 cm^2^) of *tetA*/*tetB* genes (4.34 ± 0.75) was significantly higher than the mean concentration of tetracycline-resistant *E. coli* (2.66 ± 0.61) obtained by Petrifilm (*p* < 0.001; Figure 4B). Furthermore, a Spearman rank correlation analysis showed no significant correlation between the two methods at the individual-sample level (*r_s_* = 0.13; Figure 4D, see Table 1 for full statistics). These statistical findings indicate that the methods differ both at the population level and in terms of individual sample ranking. These results strongly suggest that the *tetA*/*tetB* gene pool detected by qPCR is not exclusively associated with culturable, tetracycline-resistant *E. coli*.

Instead, these resistance genes may be carried by other bacterial species present on the carcass skin. Indeed, the presence of *tetA*/*tetB* genes in species other than *E. coli* has been widely documented [22]. The genes may also occur within non-culturable or non-viable cells or be present as extracellular DNA not associated with any intact cell. Detection of *tetA*/*tetB* genes from non-viable cells does not necessarily reflect an actively transmissible resistance risk, but the genes may still be biologically available for transfer to viable bacteria [9], and their presence may indicate selective pressure for tetracycline resistance. In line with our findings, a similar study by Guarddon et al. [24] also observed an overestimation of *tetA*/*tetB* genes on inoculated chicken meat when using the qPCR method compared with cultivation.

To investigate potential shifts in the bacterial population composition during processing, the relationship between *E. coli* and *Enterobacteriaceae* concentrations (log_10_ CFU/g) was evaluated at the population level. In the caecal samples (*n* = 75), *E. coli* (6.74 ± 0.81) constituted the vast majority of the *Enterobacteriaceae* population (6.88 ± 0.82), with a minimal mean difference of only 0.16 log_10_ units between the two groups (Figure 4A). On the carcass skin, the bacterial load generally decreases following processing on the slaughter line [25,26].

However, in the skin swab samples (*n* = 30), the mean concentration (log_10_ CFU/1400 cm^2^) of *E. coli* (3.42 ± 0.60) was significantly lower than that of *Enterobacteriaceae* (3.92 ± 0.58) (*p* < 0.001; Figure 4B). This suggests a distinct shift in the bacterial community composition at the population level, characterized by a higher proportion of non-*E. coli* members of the *Enterobacteriaceae* family on the skin in contrast to the high proportion of *E. coli* in the caecum.

Regarding the occurrence of the tetracycline resistance genes on a population level, a distinct shift was also observed between the caecal content and the skin. Specifically, the dominance of *tetA* genes (5.73 ± 1.00 log_10_ CFU/g) over *tetB* genes (4.83 ± 1.40 log_10_ CFU/g) in the caecum (*p* < 0.001) disappeared on the skin, where *tetA* (3.76 ± 0.80 log_10_ CFU/1400 cm^2^) and *tetB* (3.90 ± 0.83 log_10_ CFU/1400 cm^2^) genes exhibited similar, equal concentrations (*p* = 0.43; Figure 4).

Taken together, these findings have practical implications for individual-sample classification. Discrepancies exceeding 0.5 log_10_ units between methods occurred in 40% (34/84) of caecal samples and 63% (27/43) of skin swab samples. For skin swab samples, the systematic bias (0.75 log_10_ units) means qPCR would mainly overestimate contamination or resistance gene burden. For caecal samples, where bias was negligible, differences could go in either direction. The potential spread of tetracycline resistance genes among bacteria persisting along the pork production chain may also increase the risk of transmission of multidrug-resistant bacteria to humans due to co-resistance to other antimicrobial classes, such as β-lactams [3,27,28]. Human infections caused by multidrug-resistant bacteria are more difficult to treat, and surveillance of specific resistance genes may therefore be a valuable tool for monitoring and reducing their transmission. However, by demonstrating that qPCR cannot simply replace cultivation at the individual-sample level, despite good agreement at the population level, this study highlights the need for careful interpretation of qPCR-based resistance data and shows where qPCR can and cannot replace cultivation.

## 3. Material and Methods

### 3.1. Sampling of Pig Caecal Content and Carcass Skin Swabs

Caecal content (~35 g) from slaughter pigs (*n* = 89) and skin swab samples prior to carcass chilling (*n* = 68) were collected across four Danish abattoirs between 2015 and 2018. Both caecal and skin swab samples were collected in approximately equal numbers from each of the four abattoirs and were distributed evenly across the study period (2015–2018); a subset of these samples was subsequently selected for qPCR analysis. For the skin swabs, a 16-layer gauze tampon (10 × 10 cm) was moistened with 15 mL of buffered peptone water (BPW) (CM0509, Oxoid, Thermo Fisher Scientific, Basingstoke, UK) before swabbing a 1400 cm^2^ area covering the medial ham and abdomen. The samples were placed in a cooled container and transported to the laboratory of the Danish Veterinary and Food Administration (DVFA) in Ringsted, Denmark, for bacteriological analyses within 24 h of collection. Following the initial bacteriological processing, the samples were stored at −20 °C until qPCR analysis was performed.

### 3.2. Enumeration of Tetracycline-Resistant E. coli, E. coli, and Enterobacteriaceae by Petrifilm

One gram of caecal content (*n* = 89) was added to 9 mL of BPW (Oxoid A/S CM0509, Roskilde, Denmark) and vortexed thoroughly. From this 10^−1^ dilution, a 10-fold dilution series was prepared in 0.9% NaCl. Appropriate dilutions were immediately plated on Petrifilms as described below. For qPCR analysis, 1 mL of 10^−1^ dilution was stored at −20 °C.

For the 68 skin swabs, the gauze was transferred to a stomacher bag containing 85 mL of BPW and kneaded for 2 min. A 10-fold dilution series was prepared in 0.9% NaCl, and appropriate dilutions were immediately plated on Petrifilms as described below. For qPCR analysis, 10 mL of the initial dilution was stored at −20 °C.

Total *E. coli* and *Enterobacteriaceae*, as well as tetracycline-resistant *E. coli* from caecal and skin swab samples, respectively, were enumerated by plating 1 mL of appropriate dilutions on respective Petrifilms (Neogen Petrifilm Select *E. coli* SEC; Neogen Petrifilm *Enterobacteriaceae* EB, Fooddes A/S, Silkeborg, Denmark). Tetracycline-resistant *E. coli* was enumerated by adding 25 µL of a tetracycline solution (T3383, Sigma-Aldrich, St. Louis, MO, USA) to 1 mL of the sample dilution before plating to obtain a final tetracycline concentration of 64 mg/L in the SEC plates. This concentration was previously shown to yield resistance classifications on Petrifilm SEC plates (Neogen Petrifilm Select *E. coli* SEC; Neogen Petrifilm *Enterobacteriaceae* EB, Fooddes A/S, Silkeborg, Denmark) equivalent to those obtained using the standard 16 mg/L CLSI breakpoint on Mueller–Hinton II agar [29]. For method consistency, 25 µL of 0.9% NaCl was added to the dilutions for the enumeration of total *E. coli* and *Enterobacteriaceae*. Petrifilm plates were incubated at 37 °C for 16–18 h, following an established cultivation protocol based on prior experience with colony development and countability within this timeframe.

### 3.3. Extraction of DNA from E. coli Cultures and Caecal and Skin Swab Samples

The stored dilutions of caecal and skin swab samples were thawed at 5 °C before extraction of DNA by the DNeasy PowerLyzer PowerSoil Kit (12855, Qiagen, Hilden, Germany). Initially, caecal samples (0.5 mL 10^−1^ dilution) and *E. coli* overnight cultures (2 mL, 8 log_10_ CFU/mL, see below) were centrifuged for 5 min at 13,000 rpm (Eppendorf Mini Spin, Eppendorf, Hamburg, Germany), while skin swab samples (10 mL of initial dilution) were centrifuged at 10,000 rpm (Eppendorf 5810R, Eppendorf, Hamburg, Germany). The resulting pellets were resuspended in 750 µL Power Beads solution and 60 µL C1 reagent from the PowerLyzer kit. The proceeding DNA extraction followed the manufacturer’s instructions. The DNA was eluted in 100 µL and stored at −20 °C until qPCR analysis. Extraction efficiency was not assessed, as spike-and-recovery experiments would require a non-target organism, since *E. coli* is naturally present in virtually all faecal samples.

### 3.4. Preparation of qPCR Standard Curves for E. coli

The *E. coli* strains NCTC (National Collection of Type Cultures) 50078 (*tetA* and *uidA* genes) and CSH50::Tn10 (*tetB* gene) were provided by Professor René Henriksen (European Reference Laboratory for Antimicrobial Resistance (EURL-AR), Technical University of Denmark). These *E. coli* strains were cultured overnight in Brain Heart Infusion broth (Oxoid, Thermo Fisher Scientific, Basingstoke, UK) at 37 °C and the obtained cell concentration (CFU/mL) was determined by plate spreading of a 10-fold dilution series onto BHI agar (CM1136) containing 16 µg/mL tetracycline (T3383, Sigma-Aldrich, St. Louis, MO, USA). DNA was extracted from the overnight *E. coli* cultures (~8 log_10_ CFU/mL) as described above, followed by 10-fold dilutions of the DNA in MilliQ water for qPCR analysis of the *uidA*, *tetA*, and *tetB* genes, respectively, as described below.

Dilutions ranging from 1.6 to 7 log_10_ CFU/mL were included in each qPCR run, and the resulting measurements were pooled across runs to generate a single standard curve per gene. For each dilution, the threshold cycle (Ct) value, defined as the PCR cycle at which a sample’s fluorescence signal exceeded the baseline threshold, was plotted against the estimated concentrations (CFU/mL) of *E. coli* (*uidA* gene) and tetracycline-resistant *E. coli* (*tetA*, and *tetB* genes). Quantification was based on absolute quantification using external standard curves; no reference or housekeeping gene was used for normalization. The Ct threshold was set manually, using the same positive control (1·10^6^ CFU/mL) in every run to ensure consistent threshold placement across runs. In total, 99, 87, and 38 measurements obtained across multiple qPCR runs were used to construct the standard curves for the *uidA*, *tetA*, and *tetB* genes, respectively. The number of measurements differed between assays because they were performed in different qPCR runs during method development. All available measurements were included to improve the robustness of the regression analyses.

Linear regression analyses were then applied to convert Ct values into CFU. Dilution factors were accounted for to normalize any differences in testing volume between cultivation and qPCR methods. Based on the slope of the linear regression analysis of the standard curve, the qPCR amplification efficiency (E) was determined using the following equation: E = (10^(−1/slope)^ − 1) × 100% [30]. While ideal efficiency is 100%, values between 90% and 110% are generally considered acceptable. The 95% confidence intervals for the amplification efficiencies were calculated from the corresponding 95% confidence intervals of the regression slopes.

### 3.5. qPCR for Quantification of E. coli and Tetracycline Resistance Genes

*E. coli* quantification by qPCR was based on the *uidA* gene, encoding beta-glucuronidase [31]. For qPCR quantification of tetracycline resistance, *tetA* and *tetB* were selected as target genes, as they represent the most prevalent tetracycline resistance determinants in *E. coli* from pigs [6,32]; *tetM* was also quantified during method development but was not included in the present analysis. The primers and probes used in this study are listed in Table 2 [14,33,34]. Quantification of *E. coli* by qPCR analysis (*uidA* gene) was performed on 89 pig caecal and 68 skin swab samples in total. For the quantification of tetracycline resistance genes (*tetA* and *tetB*) by qPCR, only samples where tetracycline-resistant *E. coli* was measurable on Petrifilms were included, i.e., in total 75 caecal and 30 skin swab samples.

For the qPCR analysis, the master mix contained: 1× TaqMan^Tm^ Environmental Master Mix (4396838, Applied Biosystems, Thermo Fisher Scientific, Foster City, CA, USA), 0.5 µM forward and reverse primers (LGC Biosearch, Lystrup, Denmark), and 0.2 µM probe (LGC Biosearch, Lystrup, Denmark). The total reaction volume was 25 µL with 5 µL of sample DNA. Negative controls were added sterile water instead of template DNA. Samples were set up in a PCR 96-well plate (Mx3000P non-skirted plate in addition with Mx3000P Optical Strip Caps, Agilent Technologies, Santa Clara, CA, USA). The real-time qPCR was run on a Strategene MX3005P (Agilent Technologies, Santa Clara, CA, USA) and the thermocycler conditions were as follows: 95 °C for 10 min for activation of the polymerase; 45 cycles of 95 °C for 15 s and 60 °C for 1 min. All qPCR reactions were run in duplicate, with negative controls (sterile water) and a positive control included in each run. The positive control was a standard with a known concentration (1·10^6^ CFU/mL) specific to the target gene being analysed (*uidA*, *tetA*, or *tetB*).

### 3.6. Statistical Analysis

All cultivation data were log_10_-transformed to approximate a normal distribution, while qPCR data were quantified using standard curves based on the mean of duplicate Ct values and expressed as log_10_ CFU/g for caecal samples and log_10_ CFU/1400 cm^2^ for skin swab samples.

To evaluate mean differences on a population level, paired *t*-tests were conducted to compare (1) *E. coli* concentrations quantified via Petrifilm cultivation versus qPCR; (2) *E. coli* versus *Enterobacteriaceae* concentrations from Petrifilm cultivation; and (3) qPCR resistance gene concentrations versus tetracycline-resistant *E. coli* cultivation counts. Statistical significance was defined as *p* < 0.05. Sample sizes were determined by the scope and practical limitations of the parent surveillance project rather than an a priori power calculation.

Bland–Altman analysis was performed to assess method agreement between *E. coli* cultivation and *uidA* qPCR on an individual sample level for both caecal and skin swab samples [35,36]. The analysis was performed in Microsoft Excel. For each sample, the mean of the two methods and their difference were determined to calculate the overall mean difference (bias), standard deviation, and 95% Limits of Agreement. Only quantifiable skin swab samples on Petrifilm and the corresponding qPCR values were used to calculate the bias and limits of agreement, which therefore reflect method agreement only within this quantifiable range. Also, 95% confidence intervals for the bias and limits of agreement were calculated as bias ± 1.96 × SE, where SE is the standard error, calculated as SE(bias) = SD/√*n* and SE(LoA) = SD × √(3/*n*) [36].

The correlation between qPCR quantification of *tetA*/*tetB* genes and tetracycline-resistant *E. coli* cultivation counts within individual samples was evaluated using Spearman’s rank correlation. Significance for the correlation coefficient (*r_s_*) was established at *p* < 0.05. The 95% confidence interval for Spearman’s rank correlation coefficient (*r_s_*) was calculated using Fisher’s z-transformation. All statistical analyses were performed using Microsoft Excel.

## 4. Conclusions

For skin swab samples, the qPCR assay systematically overestimated concentrations of both *E. coli* and tetracycline-resistant *E. coli.* This is most likely due to a higher proportion of dead or non-culturable bacteria on the carcass skin compared with caecum, which implies that qPCR is less suitable for the direct quantification in skin swab samples.

For caecal samples, the qPCR assay can be used on a population level for quantification of *E. coli* and tetracycline-resistant *E. coli* but cannot replace culture-based enumeration on an individual sample level. Nevertheless, overall estimation of the presence and level of *tetA/tetB* genes remains a valuable tool to verify whether tetracycline-resistant bacteria are or have been present. This is relevant because free plasmids may also pose a public health concern, although the present study did not distinguish between cell-associated and extracellular resistance genes. Our findings support recent recommendations that molecular surveillance should complement conventional bacteriological surveillance within integrated One Health antimicrobial resistance monitoring programmes [5], and we therefore suggest that qPCR should be used as a fast first-line screening tool to monitor trends in resistance gene occurrence over time. Within such programmes, qPCR results are best used as indicators of relative resistance gene burden at the population level, rather than as precise measures of viable, culturable resistant bacteria in individual samples.

## Figures and Tables

**Table 1:** Mean concentrations of *tetA*/*tetB* genes (qPCR) and tetracycline-resistant *E. coli* (Petrifilm), and Spearman rank correlation between the two methods at the individual-sample level, for caecal (log_10_ CFU/g) and skin swab (log_10_ CFU/1400 cm^2^) samples.

	Mean Concentration	*r_s_*	95% CI	*p* (Spearman)	*n*
Caecal *					
*tetA/tetB* genes	5.91 ± 0.93	0.091	−0.14 to 0.31	0.44	75
*E. coli* tet	5.91 ± 0.86				
Skin swab **					
*tetA/tetB* genes	4.34 ± 0.75	0.13	−0.24 to 0.47	0.48	30
*E. coli* tet	2.66 ± 0.61				

* log_10_ CFU/g; ** log_10_ CFU/1400 cm^2^.

**Table 2:** PCR primers and probes for *uidA* and tetracycline resistance genes.

Gene	Primer/Probe	Sequence 5′ to 3′	Reference	Amplicon (bp)
*uidA*	F-UidA	CGGAAGCAACGCGTAAACTC	[34]	90
	R-UidA	TGAGCGTCGCAGAACATTACA		
	P-uidA	FAM-CGCGTCCGATCACCTGCGTC-TAMRA		
*tetA*	F-tetA	TTGGCATTCTGCATTCACTC	[14]	125
	R-tetA	GAAGGCAAGCAGGATGTAGC		
	P-tetA	FAM-GATCACCGGCCCTGTAGCCG-BHQ		
*tetB*	F-tetB	TTACGTGAATTTATTGCTTCGG	[33] [14]	206
	R-tetB	ATACAGCATCCAAAGCGCAC		
	P-tetB	FAM-CGCCGACCAAATCGGTCAGA-BHQ-1		

F: forward primer, R: reverse primer, P: probe.

## Data Availability

The original contributions presented in this study are included in the article. Further inquiries can be directed to the corresponding author.
